# Giant Penile Lymphedema Caused by Chronic Penile Strangulation with Rubber Band: A Case Report and Review of the Literature

**DOI:** 10.1155/2018/8598195

**Published:** 2018-05-14

**Authors:** Shunsuke Hori, Yozo Mitsui, Hidenori Iwai, Toshihiro Tai, Hideyuki Kobayashi, Koichi Nakajima, Koichi Nagao

**Affiliations:** Department of Urology, Faculty of Medicine, Toho University, Tokyo 143-8540, Japan

## Abstract

We treated a 65-year-old Japanese man with a giant penile lymphedema due to chronic penile strangulation with a rubber band. He was referred to our hospital with progressive penile swelling that had developed over a period of 2 years from chronic use of a rubber band placed around the penile base for prevention of urinary incontinence. Under a diagnosis of giant penile lymphedema, we performed resection of abnormal penile skin weighing 4.8 kg, followed by a penile plasty procedure. To the best of our knowledge, this is only the seventh report of such a case worldwide, with the present giant penile lymphedema the most reported.

## 1. Introduction

A penoscrotal lymphedema, also termed elephantiasis, results from obstruction, aplasia, or hypoplasia of lymphatic vessels. In addition to a filarial infection caused by* Wuchereria bancrofti*, the most common etiology of secondary penoscrotal lymphedema, several conditions are known to be causative. In general, penile strangulation or incarceration by a foreign body, an uncommon urological emergency condition, rarely causes penoscrotal lymphedema because most cases are treated at an early stage [1–4]. We encountered a very rare case of giant penile lymphedema in a Japanese man due to chronic penile strangulation with a rubber band.

## 2. Case Presentation

A 65-year-old single Japanese man was referred to our hospital with tremendous enlargement of the penis. There was no particular psychiatric or family history, and the patient had never travelled to a tropical region. Approximately 4 years prior to admission, he began to roll a commercially available rubber band (4.5 cm in diameter) around the penile root and foreskin, and used it throughout the day and evening to prevent urinary incontinence. Two years after beginning such rubber band use, the patient noticed gradual swelling and deformity of the penis, but continued to use the rubber band, resulting in progressive enlargement.

**Figure 1:**
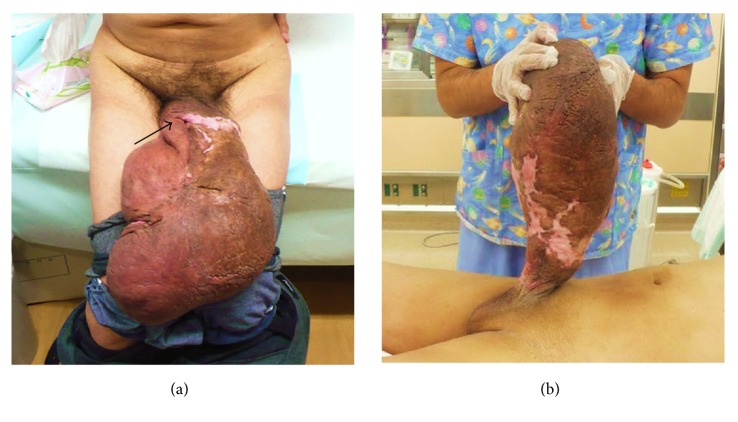
(a) Giant hypertrophic skin completely covering the penis. Black arrow indicates penile orifice. (b) Preoperative view of patient with giant penile lymphedema and normal scrotum.

A physical examination revealed brown colored hypertrophic skin that was very large (44 cm in length, maximum circumference 50 cm) though painless and completely covering the penis, with only the penile orifice barely observable (Figure 1(a)). A normal scrotum and testes could be identified after lifting the mass (Figure 1(b)). The thickened skin had no ulceration or infection, though loss of sensation for urination and erection were noted. Magnetic resonance imaging of the genital area showed an intact penis covered with edematous soft tissue. Most laboratory findings including filarial worm egg inspection were within normal limits, though urinalysis confirmed pyuria. Based on these findings, we diagnosed secondary giant penile lymphedema due to long-term chronic penile strangulation from use of a rubber band.

**Figure 2:**
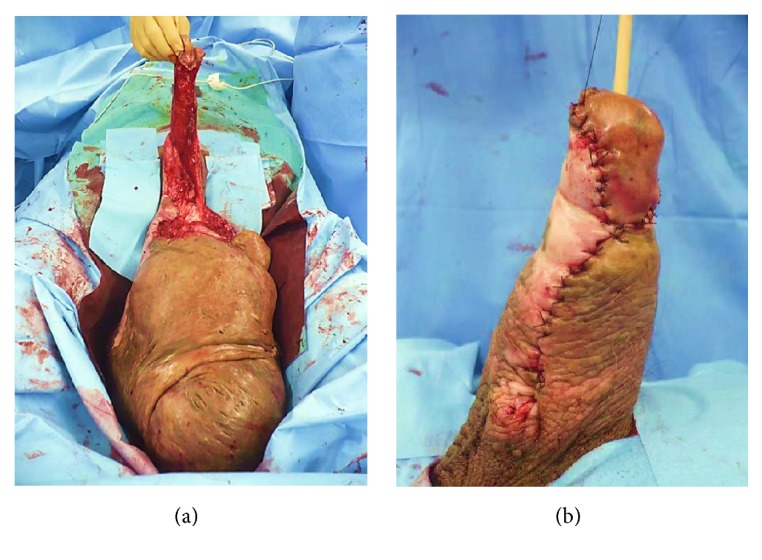
(a) Intraoperative view of giant penile lymphedema. Note the corpus cavernosa and skin near the shaft of the penis remained intact. (b) Postoperative results. Note that skin from near the penile shaft was used to cover the penis.

Surgical treatment was performed to improve quality of life (QoL) and cosmetic issues. After placing a Foley urethral catheter, surgical excision of the subcutaneous hypertrophic tissues was made with carful haemostasis to expose the corpus cavernosa, that was found to be not deformed, while skin near the shaft of the penis was also found to be normal and used to cover and preserve the penis (Figure 2(a)). Finally, we removed the infiltrated tissue (4.8 kg, 30 × 21 × 10 cm) and penile skin reconstruction was performed using absorbable sutures (Figure 2(b)).

A histopathological examination revealed that most of the excised mass consisted of proliferated collagen fibers, as well as enlarged lymphatic and capillary vessels with lymphocyte infiltration. Two months after surgery, wound healing along with acceptable cosmetic results and improvement of QoL was noted, though the patient required continuous use of a Foley urethral catheter because of loss of sensation to urinate.

## 3. Discussion

Penoscrotal lymphedema due to chronic penile strangulation or incarceration by a foreign body is extremely rare, with only 6 cases reported [1–4]. The present is the seventh case of penile lymphedema due to chronic penile strangulation described, which developed from continuous use of a rubber band around the penile base for prevention of urinary incontinence. Surprisingly, the resected abnormal huge penile skin weighed 4.8 kg and to the best of our knowledge is the largest penile lymphedema reported worldwide.

Primary and secondary forms, based on etiology, are known, with the secondary form more common. The major cause of secondary penoscrotal lymphedema is a filarial infection by* Wuchereria bancrofti*; thus it is extremely rare outside of endemic filariasis countries including Japan [5, 6]. However, it should be noted that a variety of conditions, including genital infection, inflammation, surgery, malignancy, and radiation therapy, can occasionally lead to the disease. The main mechanism underlying secondary penoscrotal lymphedema is obstruction of lymphatic vessels. Our investigation indicated no cause other than long-term usage of a rubber band in the present case; thus we speculated continuous lymphatic obstruction caused by chronic penile strangulation. Lymphatic vessels and subcutaneous tissues of the external genitalia gradually enlarged, which finally resulted in permanent histological change. Indeed, the resected tissue possessed a large number of enlarged lymphatic and capillary vessels, as well as proliferated collagen fibers, compatible with fibrosis associated with chronic lymphedema.

Application of constricting objects, such as metallic rings or rubber bands, to the penis often causes penile strangulation or incarceration and leads to painful swelling, skin ulcerations, and necrosis. Treatment in the initial stages generally consists of removal of the object and subsequent repair of any damage. However, surgical treatment is required in chronic cases such as the present, because of many problems that develop in association with hygiene, urinary incontinence, aesthetic appearance, and loss of libido, which may lead to social isolation and impaired QoL. The patients in 5 of the previously reported cases were treated with a penectomy and/or circumcision [1, 2, 4], while the remaining patient underwent resection of hypertrophic genital skin followed by covering with a skin graft [3]. In cases with extensive penoscrotal lymphedema, anatomic reconstruction with a skin flap following complete excision of all hypertrophic tissue is generally indicated [7–9]. As for the present patient, we resected most of the infiltrated tissue, with the resected specimen weighing 4.8 kg. However, the scrotum and skin of the penile shaft were normal; thus skin grafting was not required for penile skin reconstruction. Despite our finding that loss of sensation for urination was not improved at 2 months after surgery, the patient reported satisfaction with improvement of QoL.

Application of constricting objects over the penis is generally done for enhancement of sexual pleasure or curiosity [10] though it can occasionally cause penile strangulation or incarceration requiring emergency treatment. Most urologists will likely experience penile strangulation or incarceration cases during their careers; thus the possibility of development of a giant penoscrotal lymphedema when the strangulation period is extended must be considered.
